# Anxiety and the severity of Tension-Type Headache mediate the relation between headache presenteeism and workers’ productivity

**DOI:** 10.1371/journal.pone.0201189

**Published:** 2018-07-19

**Authors:** Lucas Monzani, Rosario Zurriaga, Gemma Victoria Espí López

**Affiliations:** 1 Ivey Business School at Western University, London, Canada; 2 University Research Institute of Human Resources Psychology, Organizational Development and Quality of Work Life (IDOCAL), University of Valencia, Valencia, Spain; 3 Department of Physiotherapy, University of Valencia, Valencia, Spain; Harvard Medical School, UNITED STATES

## Abstract

The primary objective of this study was to explore the mechanisms and conditions whereby Tension-Type Headache (TTH) presenteeism relates to health-related loss of productivity as a result of both reduced physical and mental health. To this end, Structural Equation Modeling (SEM) was used to conduct a secondary data analysis of a randomized clinical trial involving 78 Tension-type Headache (TTH) patients. The results showed that TTH presenteeism did not directly relate to health-related loss of productivity, either due to physical, or mental health problems. However, through anxiety-state, TTH presenteeism decreased patients’ productivity, as consequence of reduced physical and mental health. Moreover, by increasing the severity of the Tension-Type Headache, TTH presenteeism indirectly decreased patients’ productivity as consequence of reduced physical health (but not mental health). Finally, our results show that such indirect effects only occur when the cause of TTH is non-mechanical (e.g., hormonal causes, etc.). Our work provides an integrative model that can inform organizational behaviorists and health professionals (e.g., physiotherapists). Implications for organizational health are discussed.

## Introduction

Presenteeism, defined as “attending work when ill” [1], costs organizations up to $150 billion/year just in the US alone [2–4]. Moreover, presenteeism can exacerbate pre-existing physical and psychological problems and lead to workers’ long-term disability. Therefore, understanding presenteeism is critical for HR scholars and health practitioners concerned with health-related loss of productivity [5–7].

The growing interest in presenteeism emerged from two primary sources, (a) European management and health-related scholars and (b) North-American medical scholars focusing on occupational medicine. However, these two “camps” seem to have a diverging understanding of what presenteeism is (and what is it not)[1]. Whereas occupational MDs see reduced productivity as an indicator of workers’ presenteeism [8], organizational behaviorists see presenteeism as a dysfunctional behavioral pattern related to (but distinct from) health-related loss of productivity [9]. Similarly, regarding health-related loss of productivity, whereas prior studies in occupational medicine focused on productivity losses that result from reduced physical health, organizational behaviorists focused on the loss of productivity due to reduced mental health. Further, few studies simultaneously explored if specific presenteeism behaviors might relate to productivity losses that result from *both* reduced physical and mental health [7]. Such endeavor raises some compelling questions. For example, would attending work while suffering a headache (henceforth, headache presenteeism) reduce both physical and mental health directly, or might its effect be carried through mediating mechanisms? If so, which are the boundary conditions for such indirect effects? To answer these questions, the primary objective of this study is to establish if headache presenteeism relates to productivity losses due to reduced physical and mental health. However, given that International Headache Society distinguishes between several types of headaches (e.g., migraine, tension-type, etc.)[10], a more granular approach to headache presenteeism is necessary. This study focuses on tension-type headache (TTH), given that TTH is one of the most prevalent headache types within the general population [10–12], In short, TTH causes a chronic, diffuse headache pain that results from increased muscular tension in both the neck and back (pericraneal) muscles. More importantly, to answer the above questions, in this study aims to untangle the physiological and psychological mechanisms whereby TTH presenteeism might reduce productivity. For example, prior studies show that workers’ anxiety not only antecedes loss of productivity but is also an outcome of presenteeism [6,13,14]. Similarly, Campo and Darragh (2012) associated presenteeism to musculoskeletal disorders (MSD), which in turn, rank among the most frequent physiological causes of reduced productivity [15,16]. These findings are relevant to this study because Tension-Type Headache is usually defined as a particular type of MSD [12]. Thus, unpacking how the severity of the pain caused by a TTH (henceforth, TTH severity) affects workers’ productivity matters for organizations, as the prevalence of TTH in organizations has increased in recent years [17].

To sum up, whereas anxiety seems to be a psychological mechanism whereby TTH presenteeism might reduce workers’ productivity, TTH severity appears as a physiological mechanism by which this last occurs. Finally, because TTH has multiple causes that vary in nature, the indirect effect of TTH presenteeism on productivity loss through TTH severity should be conditional upon TTH cause.

### Theoretical background

Presenteeism, as defined by Johns (2010), is a dysfunctional behavioral pattern that damages workers’ health by exacerbating existing health problems [1]. While much has been written about *why* employees still attend work when ill, less attention has been paid to *how* such dysfunctional behavioral pattern leads to health-related loss of productivity [18,19]. One important caveat regarding health-related loss of productivity is that when self-reported, many mental health measures used in occupational medicine studies lack the granularity seen in organizational behavior studies, i.e., reduced mental health seems isomorphic to negative affectivity, understood as feeling irritable, nervous, hostile, or afraid [20].

We posit that TTH presenteeism relates to health-related loss of productivity. Prior studies show that working while suffering headache pain reduces workers’ ability to concentrate and effectively coordinate efforts with others (productivity losses due to reduced physical health)[21]. For example, a study involving 1781 Dutch workers in a manufacturing plant showed that those workers who attended work with a Tension-type headache (TTH presenteeism), evidenced a 20.7% overall health-related loss of productivity [22]. Further, for TTH presenteeists, productivity losses might also result from a negative affective state, as negative emotions and pessimistic moods are likely to reduce workers’ ability to concentrate and coordinate efforts with others (productivity losses due to reduced mental health).

### Anxiety-state

Anxiety is a negative affective state that may have a wide array of targets, such as social situations, assessment situations, or even technology [23–25]. Regardless of its target, anxiety (state) negatively relates to task and contextual performance and positively to counterproductive work behaviors [13]. We posit that workers displaying TTH presenteeism will be more likely to suffer from anxiety, and in turn be less productive. For example, a link between presenteeism and several negative emotions and its mutual impact on health-related loss of productivity has been reported in the occupational medicine literature [1,26].

The Job Demands-Resources Model [27] may be used to explain why anxiety state could mediate the relation between TTH presenteeism and productivity loss due to reduced mental health. More precisely, TTH presenteeists might think they can perform as well as without a headache. However, TTH presenteeists will count with fewer cognitive resources to do so given, for example, that a headache reduces immediate and delayed memory [28]. Ceteris paribus, reduced resources increase a task’s demands (“doing the same work with less”) and hence decreases workers’ perceived sense of control. According to the JDR model, this combination of low resources and high demands may generate anxiety because employees may feel unable to deal with the requirements of their job, and such reduced control increases worker anxiety. A recent meta-analysis shows that as the intensity of workers’ anxiety state increase, productivity drops as result of reduced mental health [29]. Furthermore, some evidence shows that as anxiety increases so does the likelihood of distracting somatic symptoms to appear (e.g., palpitations, dizziness), decreasing productivity as result of reduced physical health [30].

### Tension-type headache severity

As it occurs with anxiety-state, prior studies show that workers’ productivity drops as a TTH’s severity increases [31]. For example, an earlier study from this dataset shows that TTH severity can decrease workers’ productivity up to a 32.68% [32]. Similarly, TTH severity has also been associated with increased negative affective states, and thus result in loss of productivity due to reduced mental health [26,30]. Based on such prior findings, we expect that as the frequency of TTH presenteeism increases, so will increase workers TTH’s severity. If workers suffering from TTH display TTH headache presenteeism behaviors (instead of resting and allowing the pericraneal muscles to recover), they will likely push their pericraneal muscles to their highest tension level and keep them at that level. This sustained pericraneal tension is likely to increase a TTH’s chronicity, and thus its severity [33,34]. In turn, a stronger TTH severity should reduce workers’ ability to concentrate and efficiently coordinate efforts with others (i.e., as result of both the headache pain and its associated adverse affective states)[21].

### Tension-type headache cause

Whereas a TTH can have several causes, one practical way to simplify their study is to band them into larger, meta-categories. In this study, we constructed a meta-category called “mechanical causes”, which collects all TTH causes in which the TTH results from applying a voluntary (or involuntary) external mechanical force to the pericraneal muscles. For example, this meta-category includes inadequate movements, a muscular strain that results from an uncomfortable working position, strong coughs, or any other type of physical activity. Similarly, a second meta-category, “non-mechanical causes”, comprises any physiological cause that involves internal, biochemical variations, or as well systemic causes that may derive from an individual’s lifestyle. Examples of non-mechanical causes include hormonal factors (e.g., natural, or produced by birth-control pills), the effect of certain foods, and so forth [21].

We expect mechanical causes of TTH to create a higher tension in pericraneal muscles than non-mechanical causes of TTH [35]. Such difference in muscular tension is of foremost importance to this study, as they might have implications for how TTH headache presenteeism relates to productivity losses due to reduced physical health. More precisely, a widespread mechanical cause of TTH is the chronical postural strain that results from working in uncomfortable positions [36]. Such postural strain stresses the pericraneal muscles to their maximum tension level, and thus sharply increases a TTH’s severity. When muscles are at their maximum tension, they cannot contract further, regardless of how frequently workers display headache presenteeism, and in consequence, a TTH’s severity cannot increase further due to muscular contraction. Thus, we do not expect postural strain to strengthen the relationship between headache presenteeism and TTH severity. By contrast, non-mechanical causes of TTH are not triggered by situational or ergonomic factors, but by instead by inner physiological or even psychological factors, such as hormonal imbalances or intense work stress [10]. Thus, even though non-mechanical causes of TTH also create muscular tension, they do not necessarily stress pericraneal muscles to their maximum tension level. Hence, when the cause of TTH is non-mechanical, the way in which workers behave becomes more relevant regarding TTH severity. More precisely, if pericraneal muscles are not at their maximum tension level, displaying headache presenteeism when suffering from a TTH, will most likely push pericraneal muscles to their maximum tension level, which in turn should increase TTH severity. For example, Qu et al. (2018) reported a significant correlation between neuroendocrine (hormonal) deficiencies and reduced cognitive functioning in chronic TTH patients [21]. In other words, TTH headache presenteeism is more likely to relate to TTH severity, and thus productivity losses as result of reduced physical health, if pericraneal muscles are susceptible of further tension, that is when the cause of TTH was non-mechanical.

### Study hypotheses

**Fig 1:**
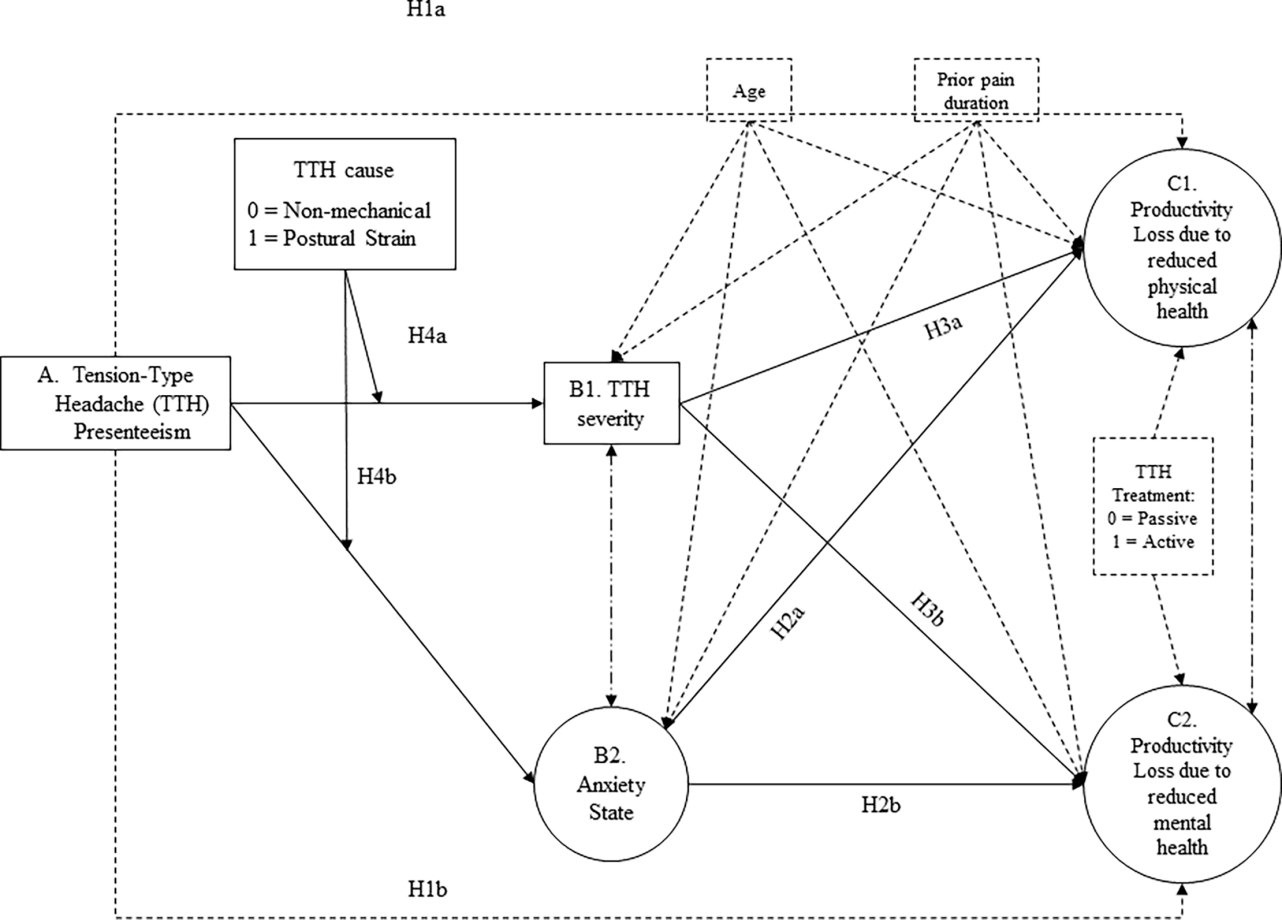
Hypothesized structural equation model relating headache presenteeism to health-related loss of productivity.

Fig 1 summarizes our theoretical model. Moreover, based on the above information, we make the following predictions:

*Hypothesis 1*: Headache presenteeism will relate to health-related loss of productivity, due to reduced (a) physical and (b) mental health.
*Hypothesis 2*: Anxiety (state) will partially mediate the relation between presenteeism and health-related loss of productivity, due to both reduced (a) physical and (b) mental health.
*Hypothesis 3*: TTH severity will partially mediate the relation between headache presenteeism and health-related loss of productivity, due to reduced (a) physical and (b) mental health.
*Hypothesis 4*: The cause of TTH (mechanical vs. non-mechanical) will moderate the indirect effect of headache presenteeism on health-related loss of productivity due to (a) physical and (b) mental problems as mediated through TTH severity. TTH severity will only mediate such indirect effect for patients whose TTH was triggered by non-mechanical causes.

## Materials and methods

We conducted a secondary analysis of a randomized clinical trial on TTH. The ethics review board of the University of Valencia (Spain) approved this study (Approval Number: H1380701837435), and the clinical trial was registered at clinicaltrials.gov with the registration number NCT02170259. Similarly, the protocol was uploaded at protocols.io and the dataset at Data Archiving and Networked Services (DANS). Participation in the clinical trial was voluntary, and each patient provided their informed consent to participate in a written document. Although the primary goal of the clinical trial differed from our objective, it is common for clinical trials to collect additional information for future use, as the specific populations they target (e.g., TTH patients) tend to be more reduced than those in organizational behavior studies.

### Sample

Of our initial sample of 80 participants, two patients were unemployed (0.25%) and thus excluded from our analyses. Our final sample consisted of 78 participants who were diagnosed with TTH in Spanish primary attention healthcare centers according to the standards of the International Headache Society [10]. All participants answered our questionnaires in full, but some declined to answer specific items. Our sample’s mean age was 39.56 (SD = 11.10). Table 1 describes our participants’ characteristics.

**Table 1:** Participants’ characteristics (N = 78).

**1. Gender assigned at Birth**	Frequency	Percentage
Male	62	79.50%
Female	19	20.50%
**2. Primary Cause of TTH**		
Postural Strain	42	53.8%
Hormonal Causes (e.g. birth-control)	28	36%
Other Causes	8	10.2%
**3. Predominant pain area**		
Occipital	26	33.3
Interparietal	30	38.5
Fronto-temporal	22	28.2
**4. Tension-Type Headache Severity**		
Mild	8	10.3
Medium	54	69.2
Medium-Moderated	16	20.5
**5. Work Sector**		
Services	41	52.56
Public administration	9	11.50
Education	11	14.10
Health	12	15.38
N/A	5	6.41
**6. Loss of Productivity (in work days per month)**	Mean	SD
Perceived overall productivity (in percentage)	67.37	20.42
Sickness absence days due to TTH	.71	2.22
Days in which performance was reduced due to TTH	8.63	6.15

Note: TTH = Tension-type Headache.

### Procedure

The clinical trial consisted of three diagnostic evaluations, and a manual therapy treatment, divided into four sessions with a seven-day interval between each session. A baseline diagnosis was conducted before any assignment to treatment conditions (pre-test), one at the end of the treatment (post-test), and one follow-up evaluation a month after the treatment (follow-up test). In each clinical evaluation, participants completed self-report questionnaires and were evaluated by certified physicians. The physician inquired about anthropometric data (age, gender), TTH causes, and evolution (headache intensity, frequency, neck and back muscular movement range, etc.). Following the baseline diagnosis, physicians scored participants’ TTH severity. At the post-treatment evaluation, participants reported their health-related loss of productivity.

Because the measures used in this study were collected at different time points, this study has a time-lagged cross-sectional design. Self-reports of TTH presenteeism refer to one month before the baseline evaluation, whereas TTH cause and severity was diagnosed by a certified physician before the treatment began (pre-test). Instead, the health-related loss of productivity scores were obtained at the post-test evaluation, that is, one month after the baseline evaluation (pre-test). All participants were randomly assigned to either active vs. passive manual therapy techniques (further details in Espí-López & Gómez-Conesa, 2010)[32].

### Measures

#### Tension-type headache presenteeism

In line with Johns' (2011) study [37], presenteeism was measured in days. More precisely, as part of the initial diagnosis, the physician asked the participants, “How many days during the last thirty days have you worked with a headache?”

#### Tension-type headache cause

As part of their initial diagnosis, the participants were asked to self-report if postural strain was the primary cause of their tensional-type headache or not, a score which was later confirmed or rejected by the physician’s diagnosis. The physician’s diagnosis of the TTH cause was dummy coded as 0 = “Non-mechanical” / 1 = “Mechanical.” Hormonal and other causes (e.g., birth-control pills) were included in the “non-mechanical” category.

#### Tension-type headache severity

The TTH’s severity was determined by a clinical interview. The interview assessed the characteristics of headaches in the month prior to the study. After the interview, physicians entered a summary score (i.e., 1 = “mild”, 2 = “medium”, and 3 = “medium-moderate”). A “mild” score represents acute, light headaches that did not have a major impact on patients’ overall physical health. A score of “medium” implied some amount of low-intensity pain prolonged in time that reduced patients’ overall health. A “medium- moderated” score, implied a frequent pain that significantly reduced the patient’s overall health.

### Anxiety (state)

We measured anxiety-state using the State-Trait Anxiety Inventory [38]. The “state” subscale measures anxiety as a temporary state through 20 questions, using a 4-item Likert scale (0 = “*not at all”* to *3 = “very much”*). Composite reliability [39] was .95 for model 3 and .94 for model 4. Example items are “I am tense” and “I am worried”.

### Health-related loss of productivity

We used four items from the Short Form 12 Health Survey (SF-HS 12) [40]. Items were scored using a Likert-type scale ranging from 1 = “Always” to 5 = “Never” and inverted when necessary. Two items (4 and 5) measured productivity losses due to reduced physical health using the following statement: “During the past four weeks, have you had any of the following problems with your work or any other regular activities as a result of your physical health?” Item 4 was “I accomplished less than I desired,” while item 5 was “I was limited in the kind of work or other activities.” Similarly, two items (6 and 7) measured Productivity losses due to reduced mental health using the following statement “During the past four weeks, have you had any of the following problems with your work or other regular activities as a result of your emotional problems”. Item 6 was “I accomplished less than I desired” and Item 7 was “I did not do work or other activities as carefully as usual.” The Composite reliability for both scales was .93.

### Data analysis

We used MPLUS 6.1 to conduct confirmatory factor analyses and structural equation models (SEM) [41]. Following Iacobucci’s recommendations [42], we choose the Robust Weighted Least Squares—Mean and Variance Adjusted (WLSMV) estimator, as this estimator deals well with non-normally distributed, ordinal data and small samples [43,44]. As goodness-of-fit indicators, besides scaled χ^2^ differences, we provide the χ^2^/DF ratio, the Root Mean Square Error of Approximation (RMSEA), Comparative Fit Index (CFI), Tucker-Lewis Index (TLI), and Weighted Root Mean Square Residual (WRMSR), taking as cut-off values a χ^2^/DF ratio below 3.86, CFI and TLI values above .90 and RMSEA and WRMSR values below .08 and 1 respectively [45–47].

In total, we constructed seven structural equation models. Models 1 and 2 test the latent factorial structure of health-related loss of productivity. In model 1, all items saturate into a single factor, whereas in model 2, items saturate into two correlated factors. To control for measurement invariance, we set similarly worded items to equality and allowed their measurement errors to co-vary. Models 3 and 4 are nested models, were Model 3 is the measurement model including all variables in this study and model 4 is a revised measurement model. We inspected items’ loadings taking .50 as cut-off criteria for retention and *p* <. 05 [48]. Instead, model 5 is our hypothesized model and model 6 reflect a more parsimonious alternative, which excludes all non-significant paths and variables. Model 7 tests the hypothesized moderated mediation by implementing Hayes’ PROCESS (2012) in MPLUS, bootstrapping 20000 subsamples to determine the parameters’ 95% *CI* [49].

### Control variables

We statistically controlled for participants’ age and the duration of participants’ existing TTH pain prior to the treatment, and the effect of the clinical trial’s treatment. The older patients are, and the longer they suffer from TTH, the more likely they are to develop a chronic strain in the pericraneal region, and for the severity of their headache to increase [50,51]. We controlled for the effect of treatment type, because prior studies on this dataset showed differences across TTH treatments [52–55]. We coded passive manual therapies with “0”, as these require a minimal intervention of physicians (e.g., myofascial inhibition and resting-state massage) and as “1”, those manual techniques that require physicians to take an active role (e.g., articulatory and combined techniques). Further, given that a connection between TTH severity and anxiety-state may also exist [56], we included a path relating anxiety-state to TTH severity in model 4 to control for their potential systematic covariation.

## Results and discussion

**Fig 2:**
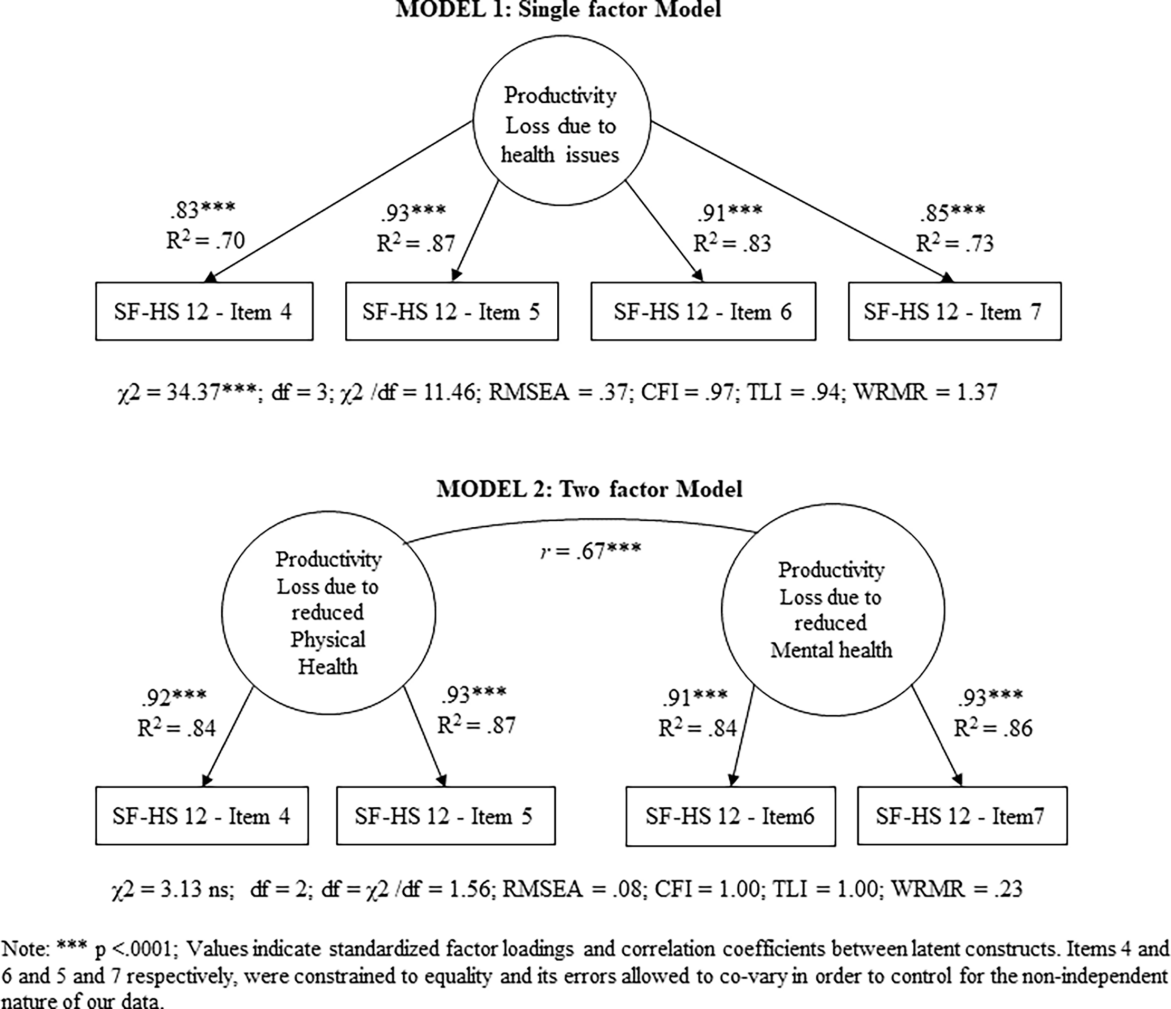
Confirmatory factor analysis for health-related loss of productivity.

Table 2 shows Means, *SD*, and Pearson’s bivariate correlations, and Fig 2 shows an illustration of factor loadings, relations and goodness-of-fit for models 1 and 2. The scaled χ2 difference test revealed that model 2 fit the data significantly better (Δχ2 = 20.77, *df* = 1, p. < .001). Similarly, the measurement model (model 3) fit our data well, and yet we optimized it in model 4. In model 4, we removed STAI’s item 11, as its loading was non-significant (β = .17, *SE* = .10; p. < .08), and the TTH Treatment variable, which was unrelated to any other variable. Table 2 shows that model 4 presented an even better fit to the data than model 3.

**Table 2:** Means, standard deviations and Pearson’s correlations (N = 78).

	Mean	SD	1.	2.	3.	4.	.5	.6	.7	.8
**1. Age**	39.56	11.10	-							
**2. Headache Presenteeism (in days)**	10.96	6.70	.06	-						
**3. TTH Cause**	.54	.50	-.04	-.08	-					
**4. TTH prior pain duration (in years)**	10.80	11.69	.32*	-.09	.22*	-				
**5. TTH Severity**	2.10	.55	-.06	.08	.32*	.25*	-			
**6. Anxiety (State)**	23.50	9.70	-.01	.23*	-.07	-.02	.14	-		
**7. TTH Treatment group**	.50	.50	-.02	-.07	-.10	.00	.05	-.04	-	
**8. Productivity loss (Physical Health)**	2.32	.87	.00	.35**	.03	.12	.28**	.37***	-.21	-
**9. Productivity loss (Mental Health)**	1.83	.79	.01	.31**	-.16	-.12	.03	.28**	-.30**	.57***

* p. < .05.

** p. < .01.

*** p. < .001.

TTH = Tensional Type Headache; TTH treatment group = 0 Non-mechanical cause / 1 = Mechanical cause

### Hypothesis testing

**Fig 3:**
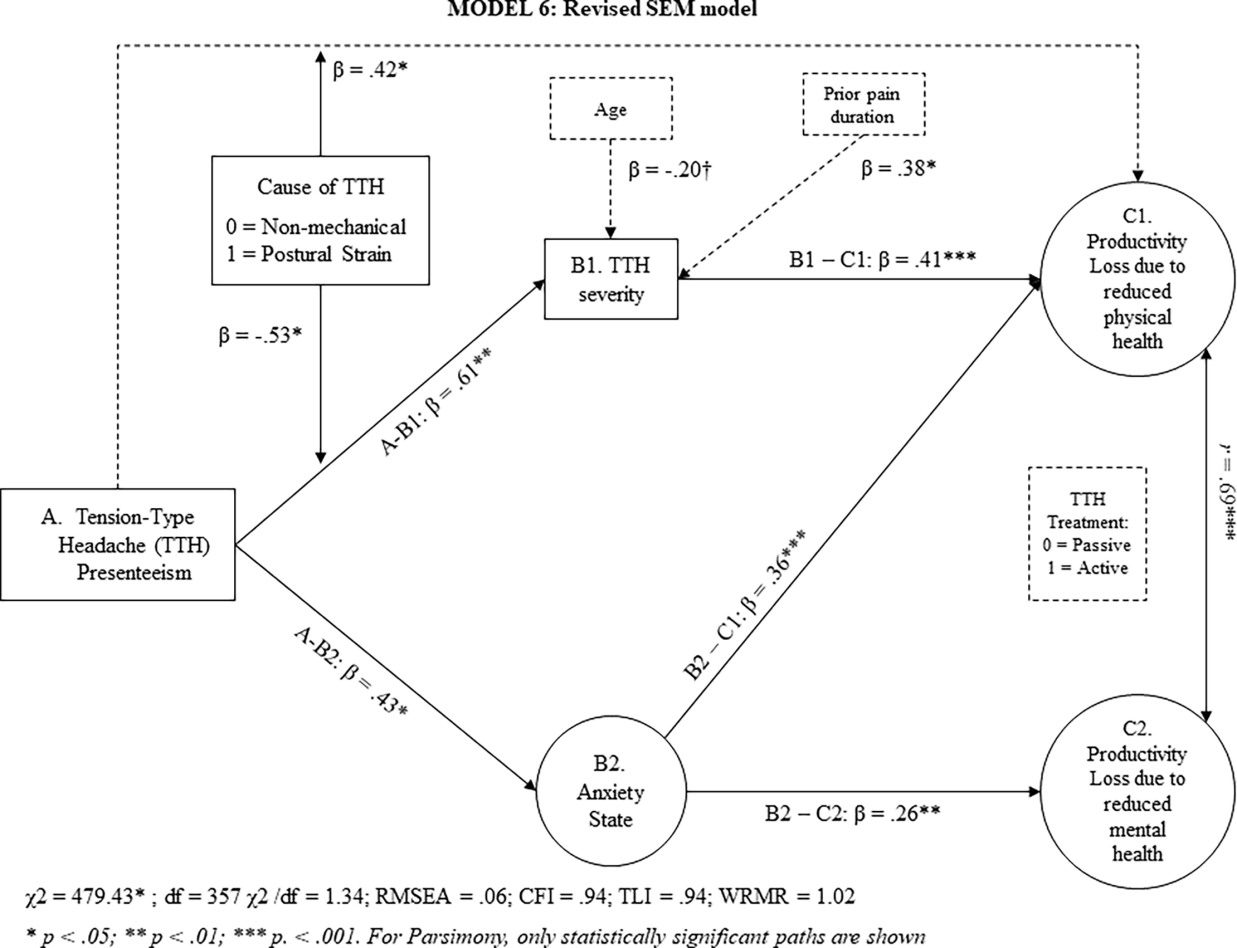
Revised structural equation model relating headache presenteeism to health-related loss of productivity.

Table 3 also shows that a more nuanced SEM (model 5) fits our data adequately. TTH presenteeism did not have a main effect on loss of productivity, neither due to reduced physical nor mental health. Thus, our results do not support hypothesis 1a or 1b. However, through anxiety-state, TTH presenteeism had an indirect effect on health-related loss productivity due to reduced physical and mental health. This result supports hypotheses 2a and 2b. Through TTH severity, however, TTH presenteeism related to loss of productivity due to reduced physical, but not mental health (see Fig 3). This result supports hypothesis 3a, but not 3b. For the sake of parsimony, any non-significant paths were removed in revised SEM model (Model 6).

**Table 3:** Confirmatory factor analysis, model fit and goodness-of-fit indicators (N = 78).

**Model**	**χ**^**₂**^	**Df**	**χ**^**₂**^**/df ratio**	**RSMEA**	**CFI**	**TLI**	**WRMR**
**Baseline Model**	1031.34***	6	171.89	-	-	-	-
**1-Factor**	34.37***	3	11.46	.37	.97	.94	1.37
**2-Factor**	3.13 *ns*	2	1.56	.08	1.00	1.00	.23
	**χ**^**₂**^	**Df**	**χ**^**₂**^**/df ratio**	**RSMEA**	**CFI**	**TLI**	**WRMR**
**Baseline model**	2536.52	465	5.45				
**Model 3**	564.78*	397	1.42	0.7	.93	.92	1.00
	**χ**^**₂**^	**Df**	**χ**^**₂**^**/df ratio**	**RSMEA**	**CFI**	**TLI**	**WRMR**
**Baseline model**	2593.34	420	6.17				
**Model 4**	513.60*	348	1.47	.08	.93	.92	1.01
**Model 5**	540.20*	370	1.46	.08	.92	.91	1.05
**Model 6**	479.43*	357	1.34	.07	.94	.94	1.02

*** *p* < .001.

** *p* < .01.

* *p* < .05.

The estimator used was a Weighted Least Squares Mean and Variance adjusted (WLSMV) with a Delta parametrization and convergence limit of 1000 interactions.

In both models 5 and 6, a significant interaction effect between TTH presenteeism and postural strain suggests that the indirect effect of TTH presenteeism on productivity loss due to reduced physical health is conditional of a TTH’s cause. Thus, a final SEM model (Model 7) tested hypothesis 4a and 4b. Bootstrapped 95% *CI* did not include zero for the indirect effect of TTH presenteeism on productivity losses due to reduced physical health as mediated through TTH severity when the TTH cause was non-mechanical (*ES* = .05, *SE* = .02; [.01, .10]). Instead, Bootstrapped 95% *CI* included zero when postural strain was the main cause of TTH (*ES* = .01, *SE* = .02; [-.04, .05]). Finally, the bootstrapped 95% *CI* for the conditional direct effect of TTH presenteeism on loss of productivity due to reduced physical health did not include zero when the cause of the TTH was postural strain (*ES* = .07, *SE* = .03; [.02, .13]). Taken as a whole, these result support hypothesis 4a but not 4b.

The primary objective of this study was to explore the mechanisms and conditions whereby tension-type headache (TTH) presenteeism relates to health-related loss of productivity. TTH presenteeism did not directly relate to health-related loss of productivity, either due to physical, or mental health problems (H1a, 1b). However, through anxiety-state, TTH presenteeism indirectly related to productivity losses as result of reduced physical and mental health (H2a, 2b). Moreover, through TTH severity, TTH presenteeism also related indirectly to productivity losses due to reduced physical health (Hypothesis 3a) but not to reduced mental health (Hypothesis 3b). However, our results reveal that such indirect relation only occurred when the cause of TTH was non-mechanical (H4).

## Discussion

Our work answers Johns’ (2010) call for an integrative and interdisciplinary study of presenteeism [1]. In this regard, a novelty of our study is that it deviates from the mainstream view of presenteeism an unspecific, generic set of behaviors, and unpacks it into different subsets presenteeism behaviors. For example, our results align with those of Wada et al., (2013) which report an association (comorbidity) between generic presenteeism, anxiety and health-related loss of productivity [26]. However, our study goes above and beyond prior studies, by showing how a specific form of presenteeism (TTH headache presenteeism) indirectly relates to workers’ health-related loss of productivity. Such unpacking opens the door for future researchers to advance the field by exploring more nuanced sets of presenteeism behaviors (e.g., Flu presenteeism, IBD presenteeism, etc.) and its linkages to more specific forms of health-related loss of productivity.

Our work provides an integrative model that is informative for organizational behaviorists and health professionals (e.g., physiotherapists). Our work aligns with current conceptualizations of presenteeism as a construct which can have a negative impact on both physical and psychological well-being. Whereas through anxiety, TTH presenteeism has a broader yet weaker effect on productivity losses, through TTH severity, TTH presenteeism has a narrower but stronger effect on productivity losses as result of reduced physical health. Moreover, by focusing on a subset of presenteeism behaviors, mainly TTH presenteeism, our findings provide key insights, but with an adequate level of granularity and specificity that contributes to design interventions aimed at reducing headache-related loss of productivity. For example, health professionals and organizational behaviorists might want to collaborate to tackle jointly the dysfunctional behaviors that result in loss of productivity due to reduced health but also to reduce the physiological factors (TTH causes) that might exacerbate such loss of productivity. Moreover, our study takes a step forward in such direction by elevating anxiety-state a critical mediating mechanism (H2a, H2b), which should not be overlooked by health professionals. Our findings also align with prior studies connecting presenteeism with Musculoskeletal disorders [16] and refine them by showing a link between a particular type of presenteeism (headache presenteeism) with a particular kind of Musculoskeletal disorder (TTH; H3a). We evidence that dysfunctional behaviors, such as TTH presenteeism, gain importance in the absence of mechanical causes of TTH. Our study has some strengths that increase the credibility of our findings. Its time-lagged, multi-source design contributes to prevent common bias in social sciences research [57,58]. Similarly, although our work is a secondary analysis of existing data, its nature, objectives, and measures do not overlap with prior studies from such dataset.

### Limitations

Our work is not without limitations. A first limitation is that this study targets a very specific population, patients suffering from TTH. Such population creates several issues that might limit the generalizability of our findings. For example, our presenteeism measure focused on a specific presenteeism behavior, mainly going to work with a chronic tension-type headache, rather than a wider range of presenteeism behaviors. Thus, the proposed mediating mechanisms (and particularly TTH severity) are very likely to be specific to TTH presenteeism. A second limitation is that our model only considers TTH severity and anxiety-state as mediating mechanisms, leaving out other potential mechanisms such as TTH frequency or intensity, which may act in parallel to TTH severity. Third, the primary study from which this study derives used a clinical interview instead of more standardized approaches to determine patients’ TTH severity, and we did not include organizational, or even psychosocial moderators for the path involving anxiety. Finally, due to the nature of our dataset, our study has a relatively small sample size in comparison to the typical survey study in organizational behavior. Such small sample size may lead to potential type II errors as result of low power. Thus, due to these limitations, we caution our readers not to overstate our findings.

Future studies should attempt to replicate our work with a larger sample, and within a real organizational setting, focusing on a wider spectrum of presenteeism behaviors, TTH causes, and use standardized measures to assess TTH severity. One possible direction could be to replicate our study involving a larger number of corporate actors (e.g., line managers), given that our work seems complementary with health-oriented leadership [59]. For example, managerial practices aimed at developing positive and psychologically safe work contexts, may deter workers’ TTH presenteeism, and thus prevent an increase in their TTH’s severity, and reduce their anxiety (and its associated decrease in productivity losses due to reduced health). For example, Monzani, Ripoll, & Peiro (2014) showed that an authentic feedback style significantly increases followers’ performance by eliciting positive emotions that counterbalance followers’ tendency towards negative affective states as result of dispositional traits (e.g., neuroticism) [60].

### Implications

TTH presenteeism leads to the reduced worker performance. As such, it increases the likelihood of avoidable errors and lower quality of service. Hence, we must surpass intervention models that focus solely on reducing absenteeism by elevating presenteeism to a similar status. HR managers should attempt to tackle health-related loss of productivity that results from tension-type headache presenteeism by combining interventions to reduce both physical (TTH severity) and emotional pain (anxiety-state). For this, besides consulting physiotherapists, including stress management techniques is paramount [61]. HR managers need to acknowledge that presenteeism matters and must understand that tackling presenteeism in all its forms adds value to their companies through the monetary benefits of a healthier and thus more productive working population.

## Supporting information

S1 Appendix Mplus scripts for all models. (DOCX)
