# Correction: Anxiety and the severity of Tension-Type Headache mediate the relation between headache presenteeism and workers' productivity

**DOI:** 10.1371/journal.pone.0202313

**Published:** 2018-08-09

**Authors:** Lucas Monzani, Rosario Zurriaga, Gemma Victoria Espí López

Fig 1 is incorrect. The authors have provided a corrected version here.

**Fig 1:**
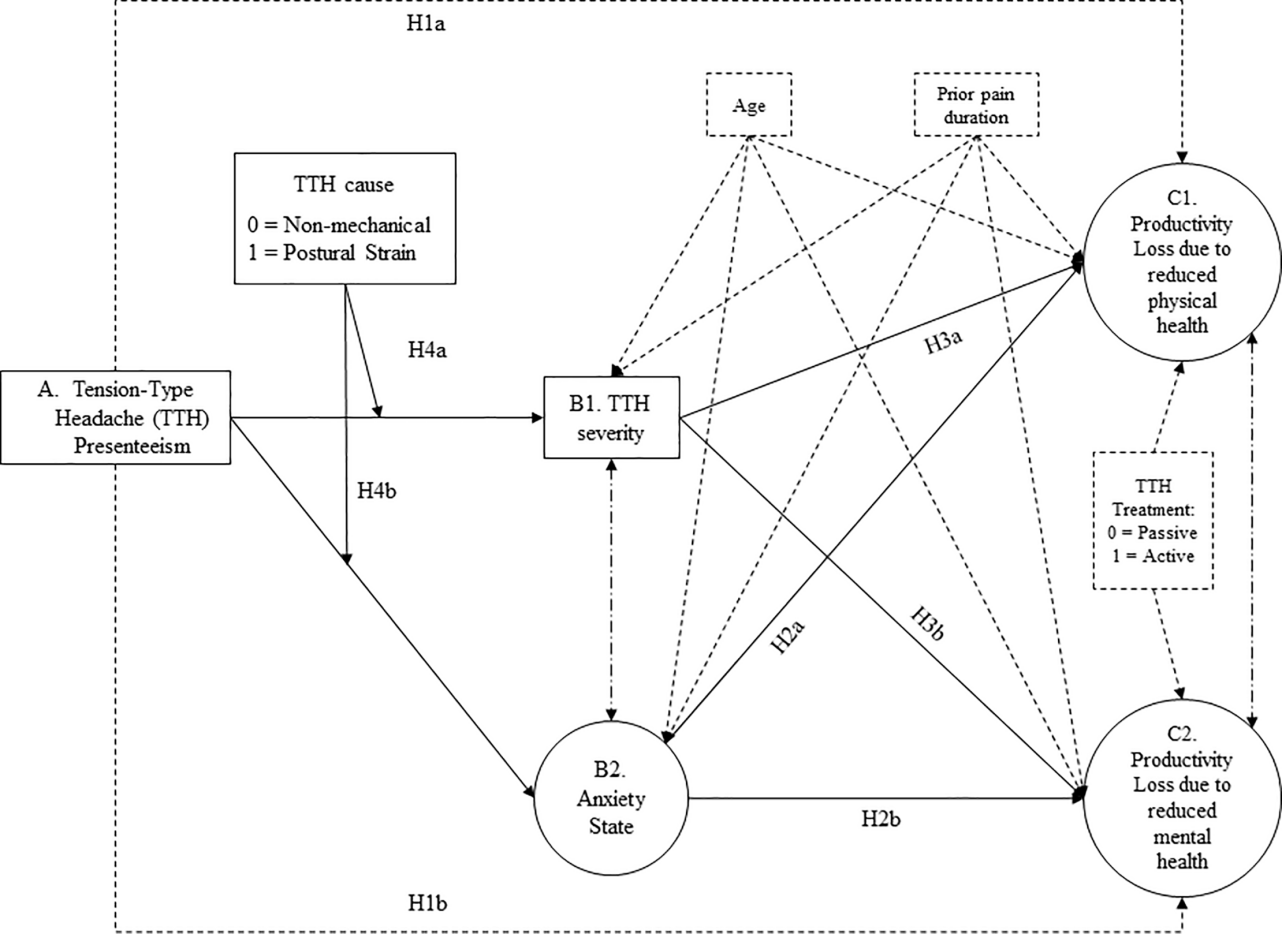
Hypothesized structural equation model relating headache presenteeism to health-related loss of productivity.
