# Supplementary material for: Anxiety and the severity of Tension-Type Headache mediate the relation between headache presenteeism and workers’ productivity
Source: PLoS One. 2018 Jul 19;13(7):e0201189. doi: 10.1371/journal.pone.0201189 (PMC6053225; doi:10.1371/journal.pone.0201189)
Supplement: S1 Appendix — (DOCX) [file pone.0201189.s001.docx]

# **S1 Appendix. Mplus scripts for all models**

| *Model 1 – Single factor model of health-related loss of productivity* |
| --- |
| *DATA:*  *FILE IS "C:\****\*****.csv"; !Filename masked to preserve anonymity*  *VARIABLE:*  *NAMES ARE Age Pres PostStr Ant PSxPres Sev*  *Stai1_i stai2_i STAi3 STAI4 sta5_i*  *STAI6 STAI7 stai8_i STAI9 STAI10*  *stai11_i STAI12 STAI13 STAI14 stai15_i*  *STAI16_i STAI17 STAI18 stai19_i stai20_i*  *AvP PL_F1 PL_F2 PL_E1 PL_E2;*  *USEVARIABLES ARE*  *PL_F1 PL_F2 PL_E1 PL_E2;*  *!Items measuring productivity loss*  *CATEGORICAL ARE*  *PL_F1 PL_F2 PL_E1 PL_E2;*  *MODEL:*  *HRLOP By*  *PL_F2 (2)*  *PL_E2 (2)*  *PL_E1 (1)*  *PL_F1 (1);*  *ANALYSIS:*  *ESTIMATOR IS WLSMV;*  *PARAMETERIZATION=DELTA;*  *ITERATIONS = 10000;*  *CONVERGENCE = 0.00005;*  *H1ITERATIONS = 5000;*  *H1CONVERGENCE = 0.0001;*  *COVERAGE = 0.10;*  *Difftest = "c:\joop\mydiff.dat"*  *OUTPUT: STDYX MODINDICES(.10);* |
|  |
| *Model 2 – Two factor model of health-related loss of productivity* |
|  |
| *DATA:*  *FILE IS "C:\****\*****.csv"; !Filename masked to preserve anonymity*  *VARIABLE:*  *NAMES ARE Age Pres PostStr Ant PSxPres Sev*  *Stai1_i stai2_i STAi3 STAI4 sta5_i*  *STAI6 STAI7 stai8_i STAI9 STAI10*  *stai11_i STAI12 STAI13 STAI14 stai15_i*  *STAI16_i STAI17 STAI18 stai19_i stai20_i*  *AvP PL_F1 PL_F2 PL_E1 PL_E2;*  *USEVARIABLES ARE*  *PL_F1 PL_F2 PL_E1 PL_E2;*  *!Items measuring productivity loss*    *CATEGORICAL ARE*  *PL_F1 PL_F2 PL_E1 PL_E2;*    *MODEL:*  *!Two factor model being “emoc”= loss of productivity due to reduced mental health and “fis” = loss of productivity due to reduced physical health*  *emoc by PL_E2(2);*  *emoc by PL_E1(1);*  *fis by PL_F2(2);*  *fis by PL_F1(1);*  *emoc WITH fis;*  *ANALYSIS:*  *ESTIMATOR IS WLSMV;*  *PARAMETERIZATION=DELTA;*  *ITERATIONS = 10000;*  *CONVERGENCE = 0.00005;*  *H1ITERATIONS = 5000;*  *H1CONVERGENCE = 0.0001;*  *COVERAGE = 0.10;*  *Savedata: Difftest is "c:\jopp\mydiff.dat";*  *OUTPUT: STDYX MODINDICES(.10);* |
|  |
| *Model 3 – Measurement model* |
|  |
| *DATA:*  *FILE IS "C:\****\*****.csv"; !Filename masked to preserve anonymity*  *VARIABLE:*  *NAMES ARE Age Pres PostStr Ant PSxPres Sev*  *Stai1_i stai2_i STAi3 STAI4 sta5_i*  *STAI6 STAI7 stai8_i STAI9 STAI10*  *stai11_i STAI12 STAI13 STAI14 stai15_i*  *STAI16_i STAI17 STAI18 stai19_i stai20_i*  *AvP PL_F1 PL_F2 PL_E1 PL_E2;*  *USEVARIABLES ARE Age Ant Pres PostStr PSxPres Sev*  *Stai1_i stai2_i STAI3 STAI4 sta5_i*  *STAI6*  *STAI7*  *stai8_i*  *STAI9 STAI10*  *stai11_i*  *STAI12 STAI13 STAI14 stai15_i*  *STAI16_i STAI17 STAI18 stai19_i stai20_i*  *AvP PL_F1 PL_F2 PL_E1 PL_E2;*  *CATEGORICAL ARE Sev PostStr AvP*  *Stai1_i stai2_i STAI3 STAI4 sta5_i*  *STAI6 STAI7 stai8_i STAI9 STAI10*  *stai11_i*  *STAI12 STAI13 STAI14 stai15_i*  *STAI16_i STAI17 STAI18 stai19_i stai20_i*  *PL_F1 PL_F2 PL_E1 PL_E2;*  *MISSING ARE ALL (-99);*  *DEFINE:*  *STANDARDIZE Age Ant Pres;*  *MODEL:*  *! Predictor variable - Pres*  *! Mediator variable(s) - STAI_E Sev*  *! Moderator variable - PSxPres*  *! Outcome variables - emoc / fis*  *! covariates - Avp / Ant*  *STAI_E by*  *Stai1_i* (l1)*  *stai2_i (l2)*  *STAI3 (l3)*  *STAI4 (l4)*  *sta5_i (l5)*  *STAI6 (l6)*  *STAI7 (l7)*  *stai8_i (l8)*  *STAI9 (l9)*  *STAI10 (l10)*  *stai11_i (l11)*  *STAI12 (l12)*  *STAI13 (l13)*  *STAI14 (l14)*  *stai15_i (l15)*  *STAI16_i (l16)*  *STAI17 (l17)*  *STAI18 (l18)*  *stai19_i (l19)*  *stai20_i (l20);*  *STAI_E@1;*  *emoc by PL_E1(plf1)*  *PL_E2(plf2);*  *fis by PL_F1(ple1)*  *PL_F2(ple2);*  *Pres with Age Ant PostStr PSxPres Sev AvP STAI_E emoc fis;*  *Age with Ant PostStr PSxPres Sev AvP STAI_E emoc fis;*  *Ant with PostStr PSxPres Sev AvP STAI_E emoc fis;*  *PostStr with PSxPres Sev AvP STAI_E emoc fis;*  *PSxPres With Sev AvP STAI_E emoc fis;*  *Sev with AvP STAI_E emoc fis;*  *AvP with STAI_E emoc fis;*  *!Composite reliability following Raykov's approach*    *MODEL CONSTRAINT:*    *NEW (RELIABSTAI); !Anxiety State (STAI)*  *NEW (RELIABFIS); !Productivity Loss due to reduced physical health*  *NEW (RELIABEMOC); !Productivity Loss due to reduced mental health*  *RELIABSTAI =*  *(l1+l2+l3+l4+l5+l6+l7+l8+l9+l10+l11+l12+l13+l14+l15+l16*  *+l17+l18+l19+l20)*  ***2/(l1+l2+l3+l4+l5+l6+l7+l8+l9+l10+l11+l12+l13+l14+l15+l16*  *+l17+l18+ l19+l20)*  ***2 + (0.292+0.506+0.178+0.311+0.595+0.323+0.746+0.780+0.476*  *+0.416+0.971+0.476+0.690+0.486+0.227+0.668+0.472+0.347+0.654*  *+ 0.534));*  *RELIABFIS =*  *(plf1+plf2)**2/(( plf1+plf2)**2+(0.140+.150));*  *RELIABEMOC =*  *(ple1+ple2)**2/(ple1+ple2)**2+(0.144+0.155));*  *ANALYSIS:*  *ESTIMATOR IS WLSMV;*  *PARAMETERIZATION=DELTA;*  *ITERATIONS = 1000;*  *CONVERGENCE = 0.00005;*  *H1ITERATIONS = 5000;*  *H1CONVERGENCE = 0.0001;*  *COVERAGE = 0.10;*  *OUTPUT: STDYX TECH4;* |
| *Model 4 – Measurement model - Revised* |
|  |
| *DATA:*  *FILE IS "C:\****\*****.csv"; !Filename masked to preserve anonymity*  *VARIABLE:*  *NAMES ARE Age Pres PostStr Ant PSxPres Sev*  *Stai1_i stai2_i STAi3 STAI4 sta5_i*  *STAI6 STAI7 stai8_i STAI9 STAI10*  *stai11_i STAI12 STAI13 STAI14 stai15_i*  *STAI16_i STAI17 STAI18 stai19_i stai20_i*  *AvP PL_F1 PL_F2 PL_E1 PL_E2;*  *USEVARIABLES ARE Age Ant Pres PostStr PSxPres Sev*  *Stai1_i stai2_i STAI3 STAI4 sta5_i*  *STAI6*  *STAI7*  *stai8_i*  *STAI9 STAI10*  *!stai11_i – Excluded item due to non-significant loading.*    *STAI12 STAI13 STAI14 stai15_i*  *STAI16_i STAI17 STAI18 stai19_i stai20_i*    *!AvP - Excluded item due to non-significant correlations*    *PL_F1 PL_F2 PL_E1 PL_E2;*  *CATEGORICAL ARE Sev PostStr*  *!AvP*  *Stai1_i stai2_i STAI3 STAI4 sta5_i*  *STAI6 STAI7 stai8_i STAI9 STAI10*  *!stai11_i*  *STAI12 STAI13 STAI14 stai15_i*  *STAI16_i STAI17 STAI18 stai19_i stai20_i*  *PL_F1 PL_F2 PL_E1 PL_E2;*  *MISSING ARE ALL (-99);*  *DEFINE:*  *STANDARDIZE Age Ant Pres;*  *MODEL:*  *! Predictor variable - Pres*  *! Mediator variable(s) - STAI_E Sev*  *! Moderator variable - PSxPres*  *! Outcome variables - emoc / fis*  *! covariates - Avp / Ant*  *STAI_E by*  *Stai1_i* (l1)*  *stai2_i (l2)*  *STAI3 (l3)*  *STAI4 (l4)*  *sta5_i (l5)*  *STAI6 (l6)*  *STAI7 (l7)*  *stai8_i (l8)*  *STAI9 (l9)*  *STAI10 (l10)*  *!stai11_i (l11)*  *STAI12 (l12)*  *STAI13 (l13)*  *STAI14 (l14)*  *stai15_i (l15)*  *STAI16_i (l16)*  *STAI17 (l17)*  *STAI18 (l18)*  *stai19_i (l19)*  *stai20_i (l20);*  *STAI_E@1;*    *fis by PL_F1(plf1)*  *PL_F2(plf2);*  *emoc by PL_E1(ple1)*  *PL_E2(ple2);*    *Pres with Age Ant PostStr PSxPres Sev STAI_E emoc fis; !AvP*  *Age with Ant PostStr PSxPres Sev STAI_E emoc fis; !AvP*  *Ant with PostStr PSxPres Sev STAI_E emoc fis; !AvP*  *PostStr with PSxPres Sev STAI_E emoc fis; !AvP*  *PSxPres With Sev STAI_E emoc fis; !AvP*  *Sev with STAI_E emoc fis; !AvP*  *!AvP with STAI_E emoc fis;*  *MODEL CONSTRAINT:*  *!Composite reliability following Raykov's approach*  *NEW (RELIABSTAI); !Anxiety State (STAI)*  *NEW (RELIABFIS); !Productivity Loss due to reduced physical health*  *NEW (RELIABEMOC); !Productivity Loss due to reduced mental health*  *RELIABSTAI =*  *(l1+l2+l3+l4+l5+l6+l7+l8+l9+l10 +l12+l13+l14+l15+l16*  *+l17+l18+l19+l20) **2/(l1+l2+l3+l4+l5+l6+l7+l8+l9+l10+l12+l13+l14+l15+l16*  *+l17+l18+l19+l20)*  ***2 +(0.293+0.505+0.179+0.313+0.594+0.322+0.746+0.780+0.477*  *+0.415 !+0.971 +0.474+0.691+0.486+0.228+0.667+0.470+0.348+0.654*  *+0.535));*  *RELIABFIS =*  *(plf1+plf2)**2/(( plf1+plf2)**2+(0.141+.150));*  *RELIABEMOC =*  *(plf1+plf2)**2/(( ple1+ple2)**2+(0.144*  *+0.155));* |
| *Model 5 – Hypothesized model* |
|  |
| *DATA*  *FILE IS "C:\****\*****.csv"; !Filename masked to preserve anonymity*    *VARIABLE:*  *NAMES ARE Age Pres PostStr Ant PSxPres Sev*  *Stai1_i stai2_i STAi3 STAI4 sta5_i*  *STAI6 STAI7 stai8_i STAI9 STAI10*  *stai11_i STAI12 STAI13 STAI14 stai15_i*  *STAI16_i STAI17 STAI18 stai19_i stai20_i*  *AvP PL_F1 PL_F2 PL_E1 PL_E2;*  *USEVARIABLES ARE*  *Age Ant*  *Pres PostStr PSxPres*  *Sev*  *Stai1_i stai2_i STAi3 STAI4 sta5_i*  *STAI6 STAI7*  *stai8_i*  *STAI9 STAI10*  *!stai11_i*  *STAI12 STAI13 STAI14 stai15_i*  *STAI16_i STAI17 STAI18 stai19_i stai20_i*  *AvP*  *PL_F1 PL_F2 PL_E1 PL_E2;*  *CATEGORICAL ARE Sev*  *Stai1_i stai2_i STAI3 STAI4 sta5_i*  *STAI6 STAI7 stai8_i STAI9 STAI10*  *STAI12 STAI13 STAI14 stai15_i*  *STAI16_i STAI17 STAI18 stai19_i stai20_i*  *PL_F1 PL_F2 PL_E1 PL_E2;*  *MISSING ARE ALL (-99);*  *DEFINE:*  *STANDARDIZE Age Ant Pres;*  *MODEL:*  *! Predictor variable - Pres (presenteeism)*  *! Mediator variable(s) - STAI_E (Anxiety State) Sev (TTH severity)*  *! Moderator variable - PSxPres (Interaction term)*  *! Outcome variables - fis (Productivity loss due to reduced physical health)*  *! - emoc (Productivity loss due to reduced emotional health)*  *! covariates - Avp (Active vs. Passive treatment) / Ant (prior pain duration)*    *!Measurement invariance - Health-related loss of productivity dimensions*  *fis by PL_F1(1)*  *PL_F2(2);*  *emoc by PL_E1(1)*  *PL_E2(2);*  *!Correlation between Health-related loss of productivity dimensions*  *fis WITH emoc;*  *! Stage 1*  *!1.1 Meditator B1: TTH severity*  *!Path A-B1 (including moderator)*  *Sev ON*  *Pres*  *PostStr*  *PSxPres*  *Age*  *Ant;*  *!1.2 Meditator B2: Anxiety-state*  *STAI_E by*  *Stai1_i stai2_i STAi3 STAI4 sta5_i*  *STAI6 STAI7 stai8_i STAI9 STAI10*  *!stai11_i*  *STAI12 STAI13 STAI14 stai15_i*  *STAI16_i STAI17 STAI18 stai19_i stai20_i;*  *!Path A-B2 (including moderator)*  *STAI_E ON*  *Pres*  *PostStr*  *PSxPres*  *Age*  *ant;*  *!Correlation between B1 and B2*  *Sev WITH STAI_E;*  *!Stage 2*  *!2.2.1 Direct effects of Headache Presenteeism and conditional effects of Postural Strain on Loss of Productivity due to Physical health*  *Fis on*  *age*  *ant*  *Pres PostStr PSxPres;*  *!2.2.2 mediation effect*  *fis ON Sev; !B1-C1*  *fis on STAI_E; !B2-C1*  *!second stage controls*  *fis on AvP;*  *!second stage controls*  *emoc on*  *AvP;*  *!Path B2-C1 & B2-C2*  *emoc on Sev; !B1-C2*  *emoc on STAI_E; !B2-C2*  *!2.2.2 Direct effects of Headache Presenteeism and conditional effects of Postural Strain on Loss of Productivity due to Mental Health*  *emoc on*  *age*  *ant*  *Pres PostStr PSxPres;*  *ANALYSIS:*  *ESTIMATOR IS WLSMV;*  *PARAMETERIZATION=DELTA;*  *ITERATIONS = 1000;*  *CONVERGENCE = 0.00005;*  *H1ITERATIONS = 5000;*  *H1CONVERGENCE = 0.0001;*  *COVERAGE = 0.10;*  *OUTPUT: MODINDICES(.10) STDYX* |
| *Model 6 – Revised Hypothesized model* |
|  |
| *DATA:*  *FILE IS "C:\****\*****.csv"; !Filename masked to preserve anonymity*  *VARIABLE:*  *NAMES ARE Age Pres PostStr Ant PSxPres Sev*  *Stai1_i stai2_i STAi3 STAI4 sta5_i*  *STAI6 STAI7 stai8_i STAI9 STAI10*  *stai11_i STAI12 STAI13 STAI14 stai15_i*  *STAI16_i STAI17 STAI18 stai19_i stai20_i*  *AvP PL_F1 PL_F2 PL_E1 PL_E2;*  *USEVARIABLES ARE*  *Age*  *Ant*  *Pres PostStr PSxPres*  *Sev*  *Stai1_i stai2_i STAi3 STAI4 sta5_i*  *STAI6 STAI7*  *stai8_i*  *STAI9 STAI10*  *!stai11_i*  *STAI12 STAI13 STAI14 stai15_i*  *STAI16_i STAI17 STAI18 stai19_i stai20_i*  *!AvP*  *PL_F1 PL_F2 PL_E1 PL_E2;*  *CATEGORICAL ARE Sev*  *Stai1_i stai2_i STAI3 STAI4 sta5_i*  *STAI6 STAI7 stai8_i STAI9 STAI10*  *STAI12 STAI13 STAI14 stai15_i*  *STAI16_i STAI17 STAI18 stai19_i stai20_i*  *PL_F1 PL_F2 PL_E1 PL_E2;*  *MISSING ARE ALL (-99);*  *DEFINE:*  *STANDARDIZE Ant Pres;*  *MODEL:*  *! Predictor variable - Pres = Headache Presenteeism*  *! Mediator variable(s)2 - SEV = TTH Severity*  *! Mediator variable(s)2 - STAI_E = Anxiety-State*  *! Moderator variable - PostStr = 0 = Non-mechanical Cause*  *! 1 = Mechanical Cause (Postural Strain)*  *! Interaction Term - PSxPres = Headache Presenteeism*  *! * Postural Strain*  *! Outcome variables - emoc = productivity loss due*  *! to reduced mental health*  *! - fis = productivity loss due*  *! to reduced physical health*  *! Covariates - Age = Participants' age*  *! - Avp = Active vs. Passive treatment*  *! - Ant = Prior pain duration*  *!STRUCTURAL MODEL - OPTIMIZED FOR PARSIMONY*  *!Measurement invariance - Health-related loss of productivity dimensions*  *fis by PL_F1(1)*  *PL_F2(2);*  *emoc by PL_E1(1)*  *PL_E2(2);*  *!Correlation between Health-related loss of productivity dimensions*  *fis WITH emoc;*  *!STRUCTURAL MODEL - OPTIMIZED FOR PARSIMONY*  *! STAGE 1*  *!Meditator B1: TTH severity*  *Sev ON Pres (a_b1);*  *Sev ON PostStr (w_b1);*  *Sev ON PSxPres (axw_b1);*  *Sev ON Age (cov2_b1);*  *Sev ON Ant (cov3_b1);*  *!Sev ON Age (cov2_b1);*  *Sev ON Ant (cov3_b1);*  *!Meditator B2: Anxiety-state*  *STAI_E by*  *Stai1_i stai2_i STAI3 STAI4 sta5_i*  *STAI6 STAI7 stai8_i STAI9 STAI10*  *STAI12 STAI13 STAI14 stai15_i*  *STAI16_i STAI17 STAI18 stai19_i stai20_i;*  *STAI_E ON Pres (a_b2);*  *STAI_E ON PostStr (w_b2);*  *STAI_E ON PSxPres (axw_b2);*  *!Removed non-significant controls:*  *!Participants Age (Age) and Prior pain (Ant:*  *!STAI_E ON Age (cov2_b2);*  *!STAI_E ON Ant (cov3_b2);*  *!Removed non-significant path: Correlation between mediators*  *!STAI_E WITH Sev;*  *! STAGE 2*  *!2.2.1 Direct effects of Headache Presenteeism and conditional effects of Postural Strain on Loss of Productivity due to Physical health*  *fis ON Sev (b1_c1);*  *fis ON STAI_E (b2_c1);*  *fis on Pres (a_c1);*  *fis on PostStr (w_c1);*  *fis on PSxPres (axw_c1);*  *!Removed non-significant controls*  *!TTH treatment (AvP), Participants Age (Age) and Prior pain (Ant)*  *!fis on AvP;*  *!fis on Age;*  *!fis on Ant;*  *!2.2.2 Direct effects of Headache Presenteeism and conditional effects of Postural Strain on Loss of Productivity due to Mental Health*  *emoc ON Sev (b1_c2);*  *emoc ON STAI_E (b2_c2);*  *!Removed non-significant predictors: TTH Causes, Headache presenteeism*  *!emoc on Pres (a_c2);*  *!emoc on PostStr (a_c2);*  *!emoc on PSxPres (axw_c2);*  *!Removed non-significant controls = TTH treatment, Participants Age (Age)*  *!Prior pain duration*  *!emoc on AvP (cov1_c2);*  *!emoc on Age;*  *!emoc on Ant*  *ANALYSIS:*  *ESTIMATOR IS WLSMV;*  *PARAMETERIZATION=DELTA;*  *ITERATIONS = 1000;*  *CONVERGENCE = 0.00005;*  *H1ITERATIONS = 5000;*  *H1CONVERGENCE = 0.0001;*  *COVERAGE = 0.10;*  *SAVEDATA:*  *DIFFTEST is "c:\jopp\SEM-dift2.dat";* |
| *Model 7 – Test of conditional indirect and direct effects* |
|  |
| *DATA:*  *FILE IS "C:\****\*****.csv"; !Filename masked to preserve anonymity*  *VARIABLE:*  *NAMES ARE Age Pres PostStr Ant PSxPres Sev*  *Stai1_i stai2_i STAi3 STAI4 sta5_i*  *STAI6 STAI7 stai8_i STAI9 STAI10*  *stai11_i STAI12 STAI13 STAI14 stai15_i*  *STAI16_i STAI17 STAI18 stai19_i stai20_i*  *AvP PL_F1 PL_F2 PL_E1 PL_E2;*  *USEVARIABLES ARE*  *Age Ant*  *Pres PostStr PSxPres*  *Sev*  *Stai1_i stai2_i STAi3 STAI4 sta5_i*  *STAI6 STAI7*  *stai8_i*  *STAI9 STAI10*  *!stai11_i*  *STAI12 STAI13 STAI14 stai15_i*  *STAI16_i STAI17 STAI18 stai19_i stai20_i*  *!AvP*  *PL_F1 PL_F2 PL_E1 PL_E2;*  *CATEGORICAL ARE*  *Sev*  *Stai1_i stai2_i STAI3 STAI4 sta5_i*  *STAI6 STAI7 stai8_i STAI9 STAI10*  *STAI12 STAI13 STAI14 stai15_i*  *STAI16_i STAI17 STAI18 stai19_i stai20_i*  *PL_F1 PL_F2 PL_E1 PL_E2;*  *MISSING ARE ALL (-99);*  *ANALYSIS:*  *ESTIMATOR IS WLSMV;*  *PARAMETERIZATION=DELTA;*  *ITERATIONS = 1000;*  *CONVERGENCE = 0.00005;*  *H1ITERATIONS = 5000;*  *H1CONVERGENCE = 0.0001;*  *COVERAGE = 0.10;*  *BOOTSTRAP = 20000;*  *DEFINE:*  *!STANDARDIZE Age; !Ant;*  *MODEL:*  *! Predictor variable - Pres = Headache Presenteeism*  *! Mediator variable(s)2 - SEV = TTH Severity*  *! Mediator variable(s)2 - STAI_E = Anxiety-State*  *! Moderator variable - PostStr = 0 = Non-mechanical Cause*  *! 1 = Mechanical Cause (Postural Strain)*  *! Interaction Term - PSxPres = Headache Presenteeism*  *! * Postural Strain*  *! Outcome variables - emoc = productivity loss due*  *! to reduced mental health*  *! - fis = productivity loss due*  *! to reduced physical health*  *! Covariates - Age = Participants' age*  *! - Avp = Active vs. Passive treatment*  *! - Ant = Prior pain duration*  *!STRUCTURAL MODEL - OPTIMIZED FOR PARSIMONY*  *!STRUCTURAL MODEL - OPTIMIZED FOR PARSIMONY*  *!Dependent Variable: Productivity loss*  *emoc by PL_E1 PL_E2;*  *fis by PL_F1 PL_F2;*  *!Correlation between latent constructs Productivity loss*  *fis WITH emoc;*  *!STRUCTURAL MODEL - OPTIMIZED FOR PARSIMONY*  *! STAGE 1*  *!Meditator B1: TTH severity*  *![Sev] (b1);*  *Sev ON Pres (a_b1);*  *Sev ON PostStr (w_b1);*  *Sev ON PSxPres (axw_b1);*  *Sev ON Age (cov2_b1);*  *Sev ON Ant (cov3_b1);*  *!Meditator B2: Anxiety*  *STAI_E by*  *Stai1_i stai2_i STAI3 STAI4 sta5_i*  *STAI6 STAI7 stai8_i STAI9 STAI10*  *STAI12 STAI13 STAI14 stai15_i*  *STAI16_i STAI17 STAI18 stai19_i stai20_i;*  *[STAI_E] (b2);*  *STAI_E ON Pres (a_b2);*  *STAI_E ON PostStr (w_b2);*  *STAI_E ON PSxPres (axw_b2);*  *! STAGE 2*  *!Productivity loss due to reduced physical health*  *fis ON Sev (b1_c1);*  *fis ON STAI_E (b2_c1);*  *fis on Pres (a_c1);*  *fis on PostStr (w_c1);*  *fis on PSxPres (axw_c1);*  *!Removed non-significant controls*  *!TTH treatment (AvP), Participants Age (Age)*  *!and Prior pain (Ant)*  *!fis on AvP;*  *!fis on Age;*  *!fis on Ant;*  *!Productivity loss due to reduced mental health*  *emoc ON Sev (b1_c2);*  *emoc ON STAI_E (b2_c2);*  *!Removed non-significant predictors: TTH Causes*  *!Headache presenteeism (main effects)*  *emoc on Pres (a_c2);*  *!emoc on PostStr (a_c2);*  *!emoc on PSxPres (axw_c2);*  *!Removed non-significant controls = TTH treatment,*  *!Participants Age (Age)*  *!Prior Pain Duration*  *!emoc on AvP (cov1_c2);*  *!emoc on Age;*  *!emoc on Ant*  *MODEL CONSTRAINT:*  *NEW(LOW_W HIGH_W*  *!Conditional Direct effect*  *D_L_C1*  *D_H_C1*  *D_L_C2*  *D_H_C2*  *!Conditional indirect effect C1*  *I_L_B1C1 I_H_B1C1*  *I_L_B1C2 I_H_B1C2*  *!Indirect effect C2*  *I_B2C1*  *I_B2C2*  *!Total effectz*  *TOT_LW1 TOT_HW1*  *TOT_LW2 TOT_HW2*  *;*  *LOW_W = 0;*  *HIGH_W = 1;*  *! Calc conditional indirect effects for each combination of moderator values*  *I_L_B1C1 = a_b1*b1_c1 + axw_b1*b1_c1*LOW_W*  *!+ cov1_c1;*  *+ cov2_b1 + cov3_b1;*  *I_H_B1C1 = a_b1*b1_c1 + axw_b1*b1_c1*HIGH_W*  *!+ cov1_c1*  *+ cov2_b1 + cov3_b1;*  *! Calc conditional indirect effects for each combination of moderator values*  *I_L_B1C2 = a_b1*b1_c2 + axw_b1*b1_c2*LOW_W;*  *!+ cov1_c2*  *!+ cov2_b1 + cov3_b1;*  *I_H_B1C2 = a_b1*b1_c2 + axw_b1*b1_c2*HIGH_W;*  *!+ cov1_c2;*  *!+ cov2_b1 + cov3_b1;*  *! Calc conditional indirect effects for each combination of moderator values*  *I_B2C1 = a_b2*b2_c1;*  *I_B2C2 = a_b2*b2_c2;*  *!Removed non-significant interaction effects*  *!I_L_B2C1 = a_b2*b2_c1*LOW_W*  *!+ cov2_b2 + cov3_b2;*  *!I_H_B2C1 = a_b2*b2_c1*HIGH_W*  *!+ cov2_b2 + cov3_b2;*  *!I_L_B2C2 = a_b2*b2_c2*LOW_W*  *!Removed non-significant control variable (age)*  *!+ cov2_b2 + cov3_b2;*  *!I_H_B2C2 = a_b2*b2_c2*HIGH_W*  *!Removed non-significant control variabe (Ant)*  *!+ cov2_b2 + cov3_b2;*  *! Calc conditional direct effects for each combination of moderator values*  *D_L_C1 = a_c1 + axw_c1*LOW_W; !+ cov1_c1;*  *D_H_C1 = a_c1 + axw_c1*HIGH_W; !+ cov1_c1;*  *!D_C2 = a_c2; !+ cov1_c2;*  *! Calc conditional total effects for each combination of moderator values*  *TOT_LW1 = I_L_B1C1 + D_L_C1;*  *TOT_HW1 = I_H_B1C1 + D_H_C1;*  *TOT_LW2 = I_L_B1C2 + D_L_C2;*  *TOT_HW2 = I_H_B1C2 + D_H_C2;*  *OUTPUT: STAND CINT(bcbootstrap);*  *!MODINDICES(.10)* |
